# Willingness to Vaccinate against COVID-19 Declines in Australia, Except in Lockdown Areas

**DOI:** 10.3390/vaccines9050479

**Published:** 2021-05-10

**Authors:** Quyen G. To, Robert Stanton, Saman Khalesi, Susan L. Williams, Stephanie J. Alley, Tanya L. Thwaite, Andrew S. Fenning, Corneel Vandelanotte

**Affiliations:** 1 Appleton Institute, Central Queensland University, Rockhampton 4701, Australia; r.stanton@cqu.edu.au (R.S.); s.khalesi@cqu.edu.au (S.K.); s.p.williams@cqu.edu.au (S.L.W.); s.alley@cqu.edu.au (S.J.A.); t.thwaite@cqu.edu.au (T.L.T.); c.vandelanotte@cqu.edu.au (C.V.); 2 School of Health, Medical and Applied Sciences, Central Queensland University, Rockhampton 4701, Australia; a.fenning@cqu.edu.au

**Keywords:** vaccine hesitancy, anti-vaccination, vaccine sentiment, pandemic, lockdown

## Abstract

This study investigates changes in willingness to vaccinate against COVID-19 and the effect of the extended restrictions in metropolitan Victoria on this change. Longitudinal and repeated cross-sectional data were collected from online surveys distributed in April, between July and August, and December 2020. Australian adults who were ≥18 years old were recruited through email lists, social media networks, and paid Facebook advertisement. Willingness to vaccinate against COVID-19 was self-reported. The results showed that participants were more willing to vaccinate if the vaccine was safe at survey 1 (longitudinal: adjusted OR (aOR) = 1.88, 95%CI = 1.38, 2.56; cross-sectional: aOR = 3.73, 95%CI = 2.55, 5.45) and survey 2 (longitudinal: aOR = 1.54, 95%CI = 1.19, 2.00; cross-sectional: aOR = 2.48, 1.67, 3.67), compared to survey 3. The change in willingness to vaccinate if the vaccine was safe and effective was not significant for those in Metropolitan Victoria; but was for those living in other Australian locations at survey 1 (OR = 2.13, 95%CI = 1.64, 2.76) and survey 2 (OR = 1.62, 95%CI = 1.30, 2.01), compared to survey 3. Willingness to vaccinate even if a vaccine had not been proven safe decreased at survey 3 (OR = 2.02, 95%CI = 1.14, 3.57) for those living in Metropolitan Victoria. In conclusion willingness to vaccinate against COVID-19 decreased over time among Australians, except for those living in metropolitan Victoria, where an additional strict and prolonged lockdown was implemented around the time of survey 2. Either the experience of the lockdown, or the presence of the COVID-19 virus itself had a positive influence on participants’ willingness to vaccinate, even if such a vaccine was not yet proven to be safe and effective.

## 1. Introduction

Vaccine hesitancy has been an issue since the development of the first vaccine. In 2019, the World Health Organization ranked vaccine hesitancy as one of the top ten threats to global health [1]. One study showed that three-quarters of American pediatricians encountered a parent who postponed or even refused for their child to receive at least one vaccine within a one-year period [2]. The percentage of non-medical vaccination exemption has also risen in the last two decades in the U.S. [3]. This is likely due to complacency and low perceived risk of infection, as studies have found vaccination rates increased in places where outbreaks occurred [4,5].

Since the World Health Organization declared COVID-19 a pandemic on 11 March 2020 [6], 219 countries and territories worldwide have reported more than 120 million confirmed cased and 2.7 million deaths (23 March 2021) [7]. In Australia, there have been 29,206 confirmed cases and 909 deaths (23 March 2021) [8]. Several vaccines have been developed and are supported for implementation globally due to evidence of their safety and efficacy (e.g., Pfizer with an efficacy of 95% [9] and AstraZeneca with an efficacy of 62.1% [10] have been approved for use in the US [11], Australia [12], and UK [13,14]). Given the severity of COVID-19, and its far-reaching implications on everyday life, one might expect that willingness to vaccinate would be high. On the other hand, given the rapid development and roll-out of COVID-19 vaccines, there may still be public concerns regarding safety and effectiveness.

Results of recent Australian studies reporting on COVID-19 vaccine hesitancy are inconsistent. One study found that the percentage of people willing to receive a COVID-19 vaccine increased 1.91% between June and July 2020 [15], whereas Alley et al. (2021) found that percentages of people willing to vaccinate decreased between April (87%) and August 2020 (85%) [16]. The disparities highlight the need to continuously track vaccine hesitancy to assist government efforts with achieving high vaccination rates.

This brief study updates the results of the Alley et al. (2021) on the changes in willingness to vaccinate for COVID-19 by including new data from a third survey conducted in December 2020. This study also aimed to investigate the impact of additional extended restrictions in the metropolitan Victoria on willingness to vaccinate for COVID-19.

## 2. Methods

### 2.1. Study Design and Participants

Australian adults (≥18 years) were invited to complete an online survey, which was created and hosted on the Qualtrics platform. Email lists, social media networks, and paid Facebook advertisement were used for recruitment. The survey was preceded by an information sheet outlining the purpose of the study and the risks and benefits of participation. Electronic consent was obtained via submission of survey responses. Three surveys were distributed, with the first one open between 9 and 19 April 2020, the second between 30 July and 16 August 2020, and the third between 1 and 25 December 2020. Participants were asked to provide an email address if they were interested in participating in future survey distribution, allowing for the collection of longitudinal data. New participants were also recruited using the same methods for the second and the third surveys. The project was approved by Central Queensland University’s Human Research Ethics Committee (Approval number 22332).

At the time of distributing the first survey, Australia was in the first wave of infection and restrictions on leaving home, travelling, and public gatherings were in place [8,17,18]. When the second survey was distributed, COVID-19 community transmission rates were very low throughout Australia, except for Victoria where rates of infection were increasing [8]. As a result, a second strict and prolonged lockdown was announced in Victoria whereas most restrictions were lifted in other states [17]. This second lockdown measures in Victoria was stricter than the first and included new measures, such as closure of childcare centres, no private gatherings, and an 8pm to 5am curfew [19]. During the third survey, rates of infection were under control, and most COVID-19 restrictions were eased in all Australian states and territories [17].

### 2.2. Measures

Demographic characteristics included age in years, gender (male, female), years of education, and weekly household income (<1000 AUD, 1000–<2000 AUD, ≥2000 AUD). Chronic disease status of participants was assessed using the question “Have you ever been told by a doctor that you have any chronic health problems?”, with response options of “yes” or “no”. Postcodes were used to classify the living location of participants as either “metropolitan Victoria” (where the lockdown occurred) or “other locations”.

Participants’ willingness to vaccinate were assessed using three questions. The first was “If a new vaccine for COVID-19 was released that was proven to be safe and effective, I would get vaccinated immediately”. The second was “If a new vaccine for COVID-19 was released but had not yet been proven to be safe and effective, I would get vaccinated immediately”; the intention of asking this question was to try to indirectly gauge whether the disruption, discomfort and potential frustration with the COVID-19 situation could entice people to vaccinate even when a vaccine has not been proven entirely safe. The third was “Even before the COVID-19 pandemic, I have always vaccinated myself against diseases when recommended by health professionals”. There were five response options “strongly disagree”, “disagree”, “neutral”, “agree”, and “strongly agree”, which were grouped into either “agree” or “disagree/neutral” for the analysis.

### 2.3. Analysis

SAS v9.4 was used for data analysis. Two datasets were created and analyzed separately. The longitudinal dataset included participants with data from at least two surveys. To ensure that the assumption of independence was not violated, the repeated cross-sectional data excluded those in the longitudinal dataset and only included participants that were different from each other for every survey. Descriptive statistics, including percentages and means with standard deviations, were generated and presented for each of the three surveys, and for the two datasets. To examine the changes in willingness to vaccinate over time, generalized linear mixed models were used for the longitudinal data, and generalized linear models were used for the repeated cross-sectional data. A binary distribution and logit link were used in both models. For each outcome, a bivariate model and a model adjusted for age, gender, years of education, household income, and chronic disease status were executed. Due to multiple comparisons, estimates were adjusted using the simulation option available in PROC GLIMMIX. Crude and adjusted odds ratios (95% confidence intervals) were reported for each outcome.

Due to the longitudinal nature of the analysis, the effect of the lockdown in metropolitan Victoria on the change in willingness to vaccinate was examined using only the longitudinal data. A generalized linear mixed model, including time, location, and an interaction term between time and location, was executed. A logit link function and binary distribution was also used. Adjustment for multiple comparisons was made using the simulation option in PROC GLIMMIX. Changes in willingness to vaccinate were presented as odds ratios for metropolitan Victoria and other locations. All *p*-values were two-tailed and considered significant if <0.05.

## 3. Results

Table 1 shows participant characteristics of both longitudinal and repeated cross-sectional samples. In general, sample characteristics were similar across all three surveys. The majority of participants were women and had household incomes of less than $2000 AUD/week. Mean age was approximately 52 years with a mean duration of education of approximately 16 years.

Table 2 shows changes in willingness to vaccinate over time. A decline in willingness to vaccinate if the vaccine was proven to be safe and effective was observed for both samples. For the longitudinal sample, participants were more willing to vaccinate at survey 1 (adjusted OR = 1.88, 95%CI = 1.38, 2.56) and survey 2 (adjusted OR = 1.54, 95%CI = 1.19, 2.00), compared to survey 3. Similar results but larger effects were found for the repeated cross-sectional sample that participants were more willing to vaccinate at survey 1 (adjusted OR = 3.73, 95%CI = 2.55, 5.45) and survey 2 (adjusted OR = 2.48, 1.67, 3.67), compared to survey 3. Although there was a decline in percentages of participants willing to vaccinate if the available vaccine had not yet been proven to be safe and effective, the difference was not statistically significant for the longitudinal sample and only significant when comparing survey 1 and survey 3 (adjusted OR = 1.75, 95%CI = 1.06, 2.89) for the repeated cross-sectional sample. Unexpectedly, the results for the question “Even before the COVID-19 pandemic, I have always vaccinated myself against diseases when recommended by health professionals” were inconsistent with longitudinal data showing a significant increase in agreement with this statement but repeated cross-sectional data showing a significant decrease in agreement.

Table 3 shows longitudinal data for changes in willingness to vaccinate by location. There was no significant difference in willingness to vaccinate if the vaccine was proven to be safe and effective for those living in Metropolitan Victoria at survey 1 and survey 2, compared to survey 3. However, a significant decrease was found for those living in other Australian locations at survey 1 (OR = 2.13, 95%CI = 1.64, 2.76) and survey 2 (OR = 1.62, 95%CI = 1.30, 2.01), compared to survey 3. Willingness to vaccinate even if a vaccine had not been proven safe and effective increased at survey 2 and decreased at survey 3 (OR = 2.02, 95%CI = 1.14, 3.57) for those living in Metropolitan Victoria. Direction of associations was not different for those living in Metropolitan Victoria and those in other locations.

## 4. Discussion

This study aimed to investigate the change in willingness to vaccinate during the COVID-19 pandemic, and the effect of extended restrictions on this change. The findings show that willingness to vaccinate declined over time in both longitudinal and repeated cross-sectional samples. This is likely due to Australia’s success in controlling the spread and mortality rates of COVID-19, hence perceived risk of infection among Australians may have reduced over time, resulting in an increase in vaccine hesitancy [4,5]. As information about perceived risk of infection was not collected, it was not accounted for in the analysis. In addition, willingness to vaccinate was higher among Australians (>85%) in April, compared to people in other countries, including France (74%) [20], New Zealand (74%) [21], the UK (64%) [22], and the USA (58%) [23], which may make it easier to decline.

Despite not being the main focus of this study, differences in the results from the longitudinal and cross-sectional data for the question “Even before the COVID-19 pandemic, I have always vaccinated myself against diseases when recommended by health professionals” was worth noting. A possible explanation for the repeated cross-sectional data could be that the samples were different for the three surveys. For the longitudinal sample, it is unclear why the percentages of agreement increased. It could be that the changes in the COVID-19 situation may somehow have influenced the way the participants responded to this question. However, given the self-report measure, the reason may just be a response bias for both longitudinal and cross-sectional data.

The results also suggest that the lockdown during survey 2 may help delay the decrease in participants’ willingness to vaccinate in metropolitan Victoria. Percentages of willingness to vaccinate even when the vaccine had not yet been proven to be safe and effective increased during the lockdown (survey 2) but then, in survey 3, they decreased to a level similar as reported in survey 1. An explanation for these changes could be that people living in places with strict lockdown conditions may be eager to have lockdown restrictions lifted and were therefore more willing to take extra measures, including vaccination (safe or not) to stop the spread of COVID-19. This result is supported by findings from a study in Italy that willingness to vaccinate against COVID-19 decreased as restrictions were relaxed [24].

To our knowledge, this is the first study investigating the possible effect of a lockdown on willingness to vaccinate against COVID-19 in a sample of Australian adults. The study included a large longitudinal sample with measures taken at times with high and low COVID-19 rates, allowing the examination of changes over time. However, there are some limitations. Survey respondents were generally older, had high levels of education, and a greater proportion were female. Therefore, generalizability may be limited to the demographics of included participants. Future studies may be needed with a sample more representative of the general Australian population. Furthermore, willingness to vaccinate was self-reported, and therefore may be subjected to recall bias. Finally, with this study design, it is not possible to control for unknown confounders.

In conclusion, willingness to vaccinate against COVID-19 decreased over time among Australians, with the exception of those living in the metropolitan Victoria where a strict lockdown was implemented around the time of survey 2. The lockdown appeared to help not only delay the decrease in willingness to receive a vaccine that was proven to be safe and effective, but also to increase willingness to vaccinate even when the vaccine had not yet been proven to be safe. Strategies to improve willingness to vaccinate among Australians are needed. Vaccination that is implemented in areas with strict restrictions may be more accepted.

## Figures and Tables

**Table 1:** Characteristics for the samples.

	Survey 1		Survey 2		Survey 3	
Longitudinal	n	% or Mean (SD)	n	% or Mean (SD)	n	% or Mean (SD)
Age (years)	638	52.5 (14.3)	858	53.2 (14.1)	560	53.5 (14.1)
Gender						
Male	199	31.3	273	31.9	165	29.6
Female	436	68.7	584	68.1	393	70.4
Years of Education	638	16.5 (4.7)	858	16.6 (4.7)	560	16.6 (4.7)
Household income/week						
<1000 AUD	148	26.5	213	28.6	142	29.3
1000–<2000 AUD	176	31.5	220	29.5	138	28.5
≥2000 AUD	234	41.9	313	42.0	205	42.3
Chronic disease						
No	335	52.5	435	50.7	271	48.4
Yes	303	47.5	423	49.3	289	51.6
Location						
Other Australian locations	550	86.2	711	82.9	453	80.9
Metropolitan Victoria	88	13.8	147	17.1	107	19.1
**Cross-Sectional**	**n**	**% or Mean (SD)**	**n**	**% or Mean (SD)**	**n**	**% or Mean (SD)**
Age (years)	1007	49.4 (15.3)	623	54.0 (15.2)	376	55.4 (14.9)
Gender						
Male	347	34.7	236	38.4	175	47.6
Female	652	65.3	379	61.6	193	52.5
Years of Education	1008	16.0 (5.3)	625	15.8 (5.4)	377	14.7 (5.4)
Household income/week						
<1000 AUD	233	27.0	192	37.0	112	38.1
1000–<2000 AUD	251	29.1	140	27.0	84	28.6
≥2000 AUD	378	43.9	187	36.0	98	33.3
Chronic disease						
No	541	53.7	314	50.2	214	56.8
Yes	467	46.3	311	49.8	163	43.2
Location						
Other Australian locations	879	87.2	443	70.9	300	79.6
Metropolitan Victoria	129	12.8	182	29.1	77	20.4

**Table 2:** Changes in willingness to vaccinate over time.

Longitudinal Data	Survey 1 (n = 638)		Survey 2 (n = 858)		Survey 3 (n = 560)		Crude OR (95% CI)		Adjusted OR (95% CI) ^+^	
	n	%	n	%	N	%	T1 vs. T3	T2 vs. T3	T1 vs. T3	T2 vs. T3
If a new vaccine for COVID-19 was released that was proven to be safe and effective, I would get vaccinated immediately										
Disagree/Neutral	82	12.9	127	14.8	118	21.1	1.00	1.00	1.00	1.00
Agree	556	87.2	731	85.2	442	78.9	1.91 *** (1.43, 2.54)	1.59 *** (1.25, 2.02)	1.88 *** (1.38, 2.56)	1.54 *** (1.19, 2.00)
If a new vaccine for COVID-19 was released but had not yet been proven to be safe and effective, I would get vaccinated immediately										
Disagree/Neutral	549	86.1	729	85.0	494	88.2	1.00	1.00	1.00	1.00
Agree	89	14.0	129	15.0	66	11.8	1.26 (0.89, 1.78)	1.38 * (1.01, 1.88)	1.19 (0.81, 1.73)	1.31 (0.94, 1.84)
Even before the COVID-19 pandemic, I have always vaccinated myself against diseases when recommended by health professionals										
Disagree/Neutral	115	18.0	127	14.8	55	9.8	1.00	1.00	1.00	1.00
Agree	523	82.0	731	85.2	505	90.2	0.57 *** (0.44, 0.75)	0.69 *** (0.54, 0.88)	0.61 *** (0.46, 0.82)	0.74 * (0.57, 0.96)
**Cross-Sectional Data**	**Survey 1 (n = 1008)**		**Survey 2 (n = 625)**		**Survey 3 (n = 377)**		**Crude OR (95% CI)**		**Adjusted OR (95% CI)**	
	**n**	**%**	**n**	**%**	**N**	**%**	**T1 vs. T3**	**T2 vs. T3**	**T1 vs. T3**	**T2 vs. T3**
If a new vaccine for COVID-19 was released that was proven to be safe and effective, I would get vaccinated immediately										
Disagree/Neutral	151	15.0	131	21.0	148	39.3	1.00	1.00	1.00	1.00
Agree	857	85.0	494	79.0	229	60.7	3.67 *** (2.65, 5.07)	2.44 *** (1.74, 3.42)	3.73 *** (2.55, 5.45)	2.48 *** (1.67, 3.67)
If a new vaccine for COVID-19 was released but had not yet been proven to be safe and effective, I would get vaccinated immediately										
Disagree/Neutral	837	83.0	529	84.6	337	89.4	1.00	1.00	1.00	1.00
Agree	171	17.0	96	15.4	40	10.6	1.72 * (1.11, 2.67)	1.53 (0.96, 2.45)	1.75 * (1.06, 2.89)	1.60 (0.95, 2.71)
Even before the COVID-19 pandemic, I have always vaccinated myself against diseases when recommended by health professionals										
Disagree/Neutral	185	18.4	131	21.0	112	29.7	1.00	1.00	1.00	1.00
Agree	823	81.7	494	79.0	265	70.3	1.88 *** (1.36, 2.61)	1.59 ** (1.12, 2.27)	1.58 * (1.07, 2.32)	1.37 (0.91, 2.07)

* *p* < 0.05, ** *p* < 0.01, *** *p* < 0.001. ^+^ Adjusted for age, gender, years of education, household income, and chronic disease status.

**Table 3:** Percentage of agreement at three surveys and changes by location for longitudinal data.

	Survey 1 (n = 638)		Survey 2 (n = 858)		Survey 3 (n = 560)		T1 vs. T3	T2 vs. T3
	n	%	n	%	n	%		
If a new vaccine for COVID-19 was released that was proven to be safe and effective, I would get vaccinated immediately								
Other Australian location	481	87.5	599	84.3	349	77.0	2.13 *** (1.64, 2.76)	1.62 *** (1.30, 2.01)
Metropolitan Victoria	75	85.2	132	89.8	93	86.9	0.99 (0.52, 1.86)	1.45 (0.82, 2.54)
If a new vaccine for COVID-19 was released but had not yet been proven to be safe and effective, I would get vaccinated immediately								
Other Australian location	75	13.6	95	13.4	52	11.5	1.24 (0.90, 1.72)	1.24 (0.92, 1.66)
Metropolitan Victoria	14	15.9	34	23.1	14	13.1	1.28 (0.64, 2.57)	2.02 * (1.14, 3.57)
Even before the COVID-19 pandemic, I have always vaccinated myself against diseases when recommended by health professionals								
Other Australian location	449	81.6	597	84.0	405	89.4	0.59 *** (0.47, 0.75)	0.66 *** (0.53, 0.82)
Metropolitan Victoria	74	84.1	134	91.2	100	93.5	0.43 ** (0.23, 0.79)	0.88 (0.50, 1.55)

* *p* < 0.05, ** *p* < 0.01, *** *p* < 0.001. The model includes time, location, and time*location.

## Data Availability

The data presented in this study are available on request. The data are not publicly available due to ethical considerations.
